# Understanding American Public Support for COVID-19 Risk Mitigation: The Role of Political Orientation, Socio-Demographic characteristics, Personal Concern, and Experience, the United States, 2020

**DOI:** 10.3389/ijph.2021.1604037

**Published:** 2021-07-01

**Authors:** Wanyun Shao, Feng Hao

**Affiliations:** ^1^ Department of Geography, University of Alabama, Tuscaloosa, AL, United States; ^2^ Department of Sociology, University of South Florida, Tampa, FL, United States

**Keywords:** COVID-19 mitigation measures, political polarization, elite cues, personal experience, structural equation modelling

## Abstract

**Objectives:** COVID-19 is the most challenging public health crisis in decades in the United States. It is imperative to enforce social distancing rules before any safe and effective vaccines are widely available. Policies without public support are destined to fail. This study aims to reveal factors that determine the American public support for six mitigation measures (e.g., cancel gatherings, close schools, restrict non-essential travel).

**Methods:** Based on a nationally representative survey, this study uses Structural Equation Modelling to reveal the relationships between various factors and public support for COVID-19 mitigation.

**Results:** 1). Democrats are more likely than Republicans to support mitigation measures; 2).Favorability towards the political leader (Biden or Trump) can slant public support for COVID-19 mitigation measures among different segments of the public.; 3). Indirect experience, rather than direct experience with COVID-19 can motivate people to support mitigation; 4). Concern for COVID-19 is a strong motivator of support for mitigation.

**Conclusion:** Political polarization poses an enormous challenge to societal well-being during a pandemic. Indirect experience renders COVID-19 an imminent threat.

## Introduction

COVID-19 has emerged as the most challenging public health crisis in decades, against the backdrop of intensifying polarization in the American public. Ever since its outbreak in the Spring of 2020, COVID-19 has infected tens of millions of Americans and caused hundreds of thousands of deaths in the United States [1]. Being confronted with this unprecedented public health crisis, the American public however is divided on various facets of COVID-19. Public response to COVID-19 seems to follow a clear political line. Liberals are more concerned about COVID-19’s impact on public and personal health, U.S. economy, one’s financial situation, and local community, compared to conservatives [2]. This public division on risk perceptions of this pandemic not only reflect in different partisanships but also in demographic characteristics [2]. In terms of race and ethnicity, nonwhites including blacks, Hispanics, and Asians perceive higher risk perceptions of COVID-19 than white people. Individuals in the lower income bracket perceive higher risk perceptions than people in upper income level.

A rapidly growing number of studies have been dedicated to reveal the geographic pattern [3] of and socio demographic differences of public response to COVID-19 [4, 5]. Some of the studies attribute the polarizing public response to COVID-19 to political orientation. In early months of the outbreak, the risk of COVID-19 was compared with the flu by some conservative personalities [6] and various conspiracy theories were widely disseminated by conservative media [7]. A recent study finds that President Trump is a major driving force of COVID-19 misinformation [8].

As COVID-19 continues to affect the entire American public, it is imperative to enforce social distancing rules and other precautionary policies before any safe and effective vaccines are widely available. Policies without public support are destined to become ineffective. How the public responds to these policies thus warrants a close examination. Although there is a rapidly growing number of studies on risk perceptions of COVID-19 based on survey data [2, 9] and social-distancing behaviors based on location data from mobile phones [10, 11], there is a paucity of studies specifically dedicated to understand public support for a comprehensive list of mitigation measures during a pandemic. Given the intense politicization of COVID-19, it is implausible to investigate public support for COVID-19 without regards to the political influence, especially during a United States presidential election year. In this study, our overarching research question is how the polarizing context influences public support for policies mitigating COVID-19 risks. Particularly, we are interested in how attitudes towards the prominent political leaders of different parties can further slant policy support among different segments of the public. Along with sociopolitical factors, we examine how the use of social media, concern for COVID-19, and experience with the disease affect public support. In addition to the policy implications, this study presents a case study that illuminates the great challenge posed by polarization on the societal well-being. In the remainder of this section, we discuss relevant literature and compose our hypotheses based on the literature.

American public polarization has been closely observed and studied in recent decades [12]. A great number of studies and surveys have identified and repeatedly confirmed this trend. As manifestations of polarization, the American public is divided on a wide range of issues such as gun control policy, abortion, racial attitudes, climate change, and immigration [13]. A common pattern that has been identified to be emblematic of polarization across countries is the reduction of multiplicity of “within-group” differences, coupled with the reinforcement of perceived intergroup differences [14]. The rhetoric “us vs. them” is thus prevalent in polarized societies. Geographical as well as sociopolitical polarization has greatly increased over the past 40 years in the United States, giving rise to fertile soil for fake news to grow [15]. Fake news further polarizes the public [15]. The popularity of social media such as Twitter among voters also contributes to increasing polarization [16]. Some scholars contend that some level of polarization can provide some benefits such as helping mobilize supporters and strengthen political parties. Unfettered polarization however can lead to growing social tension, disinclination to compromise and to achieve consensus, and ultimately weakening democratic norms and institutions [14]. In addition, polarization has given rise to increasing affective partisanship in the United States electorate [17], as witnessed in the strong emotions such as anger and frustration towards the opponents. With a pandemic raging, public health tends to fall prey to sociopolitical polarization.

Social scientists have long made the observation that public opinion at the individual level are fickle and unstable [18]. Abundant empirical evidence suggests that public views of policy issues can be largely shaped by how the issues are framed. In Framing Theory Chong and Druckman [19, 104] define framing as “the process by which people develop a particular conceptualization of an issue or reorient their thinking about an issue.” According to the authors, by choosing to emphasize certain features of a policy and neglect other features, different frames in mind can be invoked among the recipients. Empirical evidence exists to indicate the power of framing. For instance, framing hate group rally in the contexts of “freedom of speech” and “risk of violence” can lead to distinctive responses [20]. Due to the nature of multifaceted aspects of nearly every modern political issue, influential figures such as politicians and other elites (e.g., religious leaders, notable journalists, and renowned scientists) are in unique positions to frame issues as average citizens lack the time and resources that are needed to form evidence-based and thought-out judgement on all issues. Especially “when an issue is new to the agenda, the public is uncertain of its stakes and of how competing positions relate to their values. In the formative stages of an issue, opposing sides may each contend that its position is consistent with the core values and priorities of the voters it is targeting” [19, 113].

Meanwhile, despite the evidence of unstable public opinions, some social scientists find that public opinion towards various issues and policies at the aggregate level displays sensible patterns reflecting the changes in social and political conditions [21]. This apparent discrepancy is intriguing at first sight. Scholars further suggest that heuristics such as elite cues play a substantial role in turning the general ignorance among individuals to rational stances as a group [22]. When citizens are faced with a very complex issue requiring specialized knowledge and training to understand, they tend to depend on the cues of elites they deem credible and trustworthy [22]. If elite cues are powerful, from which elites are cues taken? Deriving from the theory of motivated reasoning, credibility and trust can be cultivated among like-minded people [22]. In other words, the leader who is regarded to represent people’s values and interests is embraced as one of “us” and is entrusted. Especially for scientific and technical issues, thanks to their complexities, the public is more likely to turn to political leaders they deem like-minded.

Ever since his announcement to run for the United States president, President Trump has emerged as the most influential polarizing figure in the United States politics. He is likely to evoke strong emotions among different segments of the public. Compared to conventional political conservatives, Trump embodies right-wing populism [23], as he often uses divisive rhetoric to provoke fears among “a homogeneous people” that is portrayed to be under siege by “the dangerous other” [24]. Trump’s excessive use of social media throughout his presidency (before his account was suspended by Twitter) had successfully maneuvered public attention [25] and set his own political agenda among his supporters [26]. His words and actions tended to have a deep resonance with a large segment of the public, while stirring equally strong resentment among others. On a psychological level, Trump’s explicit exploitation of racial and ethnical divisions effectively attracted a great number of white working-class voters but repel others including minorities and college educated white Americans [27]. According to the frame theory, the strength of a frame does not necessarily rest upon “intellectually or morally superior arguments” but “can be built around exaggerations and outright lies playing on the fears and prejudices of the public” (19, 111). “Fears” and “prejudices” have been the main theme running through Trump’s ascent in the political view and his presidency. Because of the strong visceral reactions evoked by Trump, it is expected that his framing of various issues and policies can profoundly shape his supporters’ views through the mechanism of elite cues. Further, the more enthusiastic one’s approval of Trump as the president is, the stronger effects his framing of issues has. The immense influence of elite cues in shaping public opinions has been found in the discourse of climate change [28]. Moreover, the effects of Trump have been documented in a study of public risk perceptions of climate change [29]. In an extremely polarized political environment, the Democratic political leaders who are perceived to be the opposition to Trump can be enthusiastically embraced by voters who resent Trump. Their words and actions can thus appeal to an equally sizable population and counteract the opposition.

When COVID-19 first broke out, many aspects of the novel coronavirus such as its origin, contagiousness, and lethality remained uncertain. A great amount of uncertainties thus gave plenty of room to elites to influence public opinion. Political leaders from either party have sent distinctive signals since the very beginning. Many conspiracy theories have been widely disseminated among conservatives, filling a void of information [30]. President Trump reportedly deliberately downplayed the risk of COVID-19 out of fear for creating a panic [31]. The mainstream media and Democratic leaders such as Biden on the other hand have incessantly emphasized the high contagiousness of the pandemic and the threat it would pose to the public health. The framing environment for COVID-19 is thus highly competitive and sometimes contradictory. Influenced by the elite cues, Republicans and Democrats have responded to this disease differently.

As influential as political leaders are, personal experience with this disease is expected to reinforce or counteract the effect of framing and elite cues. Construal Level Theory (CLT) proposes that individual perceptions and behaviors are largely influenced by one’s construed psychological distance to the subject of interest [32]. If the perceived distance to the risk is imminent and immediate in terms of both time and space, one’s risk perceptions would be heightened. Based on the Protective Action Decision Model (PADM), heightened risk perception can be conducive to risk mitigation behavioral response [33]. According to the CLT theory as well as the PADM, personal experience either directly or indirectly with COVID-19 would render this disease urgent and motivate mitigation behavioral response. Nevertheless, the effects of personal experience in risk mitigation behavioral response are inconclusive in the literature [34]. For instance, flood experience is found to have direct effects on flood mitigation *via* risk perception in one U.S. study [35]. In a study based in the south England, people who have experienced flood damages are not more likely than others to perceive and respond to climate change risk [36]. Previous earthquake experience is not found to be correlated with evacuation intention [37]. The inconsistent relationship between experience and response can be partially attributed to the difficulty of defining and measuring experience [38].

According to PADM, the immediate precursor that triggers protective action decision making is a cluster of core perceptions including threat perceptions, protective action perceptions, and stakeholder perceptions. In practice, no consensus exists on the mental models of hazards and concern is thus adopted as a global construct to represent the multidimensionality of core perceptions [33].

Based on the literature review, we propose four hypotheses:

1. Democrats are more likely than Republicans to support for COVID-19 mitigation measures.
2. Favorability towards the political leader (Biden or Trump) can slant public support for COVID-19 mitigation measures among different segments of the public.
3. Personal experience with COVID-19 can motivate people to support for COVID-19 mitigation measures.
4. Concern for COVID-19 can motivate people to support for COVID-19 mitigation measures.

## Methods

The data used in this study is the latest wave of the Nationscape survey that was conducted between June 25 and July 1, 2020 with 6,479 respondents. Nationscape is a 16-months election study on American adults (age 18 and over) carried out by researchers at UCLA and started back in July of 2019 [39]. The interviews were conducted online and in English. The samples were provided by Lucid, a market research platform that runs an online exchange for survey respondents. The samples drawn from this exchange match a set of demographic quotas on age, gender, ethnicity, region, income, and education. The survey data are then weighted to be representative of the American population according to benchmarks on the 2017 American Community Survey of the U.S. Census.

We use structural equation modeling (SEM) for statistical estimation, which helps specify a conceptual model to estimate both direct and indirect effects from exogenous variables to the endogenous variable [40, 41]. We introduce the variables below and the summary statistics are presented in Table 1.

**TABLE 1:** Descriptive statistics. Understanding American Public Support for COVID-19 Risk Mitigation: The Role of Political Orientation, Socio-Demographic characteristics, Personal Concern, and Experience, the United States, 2020.

	Mean	S.D.	Min	Max
*Endogenous Variables*				
Cancel all meetings or gatherings of more than 10 people	3.158	1.011	1	4
Close businesses where larger numbers of people gather	3.152	1.006		
Close schools and universities	3.026	1.055		
Require people who can work from home to work from home	3.423	0.833		
Restrict all non-essential travel outside the home	2.843	1.093		
Test people for a fever before entering public buildings	3.320	0.877		
*Exogenous Variables*				
Favorability of Biden as the presidential candidate	2.453	1.128	1	4
Favorability of Trump as the presidential candidate	2.269	1.258		
Political party affiliation (leaning democrats)	4.050	2.233	1	7
Political party affiliation (leaning republicans)	3.950	2.233		
Sex (Female = 1)	0.511	0.500	0	1
Age	45	17	18	93
Education	2.274	1.028	1	4
Household income	2.446	1.109	1	4
Race (White = 1)	0.743	0.437	0	1
Ethnicity (Hispanic = 1)	0.151	0.358	0	1
Religion (Evangelical = 1)	0.331	0.470	0	1
Get news from social media (e.g., Facebook, twitter, etc.) (yes = 1)	0.721	0.449	0	1
Concern about coronavirus in the United States	3.395	0.819	1	4
Direct experience with the coronavirus	0.194	0.467	0	2
Indirect experience with the coronavirus	0.583	0.693	0	2

### Endogenous Variable: Public Support of Mitigation Measures

There are six variables in the data that measure one’s level of support for a number of COVID-19 mitigation measures: 1) cancel all meetings or gatherings of more than 10 people, 2) close businesses where larger numbers of people gather, 3) close schools and universities, 4) require people who can work from home to work from home, 5) restrict all non-essential travel outside the home, and 6) test people for a fever before entering public buildings. Responses are coded in four categories including strongly oppose (1), somewhat oppose (2), somewhat support (3), and strongly support (4). A higher value indicates a more supportive stance of these measures than a lower value. The confirmatory factor analysis results in Table 2 show that the standardized factor loadings of all individual items are statistically significant, and the loadings are reasonable in magnitude (all above 0.6). The results suggest adequate reliability of using these variables to construct a latent measure of public support for COVID-19 mitigation measures. The Cronbach’s Alpha score for these six indicators is 0.892.

**TABLE 2:** Confirmatory factor analysis of public support for COVID-19 mitigation measures. Understanding American Public Support for COVID-19 Risk Mitigation: The Role of Political Orientation, Socio-Demographic characteristics, Personal Concern, and Experience, the United States, 2020.

	Standardized loadings
Cancel all meetings or gatherings of more than 10 people	0.877***
Close businesses where larger numbers of people gather	0.836***
Close schools and universities	0.803***
Require people who can work from home to work from home	0.666***
Restrict all non-essential travel outside the home	0.750***
Test people for a fever before entering public buildings	0.621***

***p < 0.001.

### Exogenous Variables

There are two variables measure how favorable respondents consider either Donald Trump or Joe Biden as the presidential candidate. Response categories include very unfavorable (1), somewhat unfavorable (2), somewhat favorable (3), and very favorable (4). A higher value indicates a greater level of favorability than a lower value.

Next, there are several variables about one’s political-socio-demographic background. Political party affiliation, ranging from 1 to 7, is coded in two ways. For one variable, a higher value means leaning to Democrats while for the other variable, a higher value means leaning to Republicans. In addition, we include variables of sex (female = 1), age, education (high school and less = 1, some college = 2, undergraduate degree = 3, and graduate and higher = 4), household income (less than *$*25,000 = 1, *$*25000–*$*49,999 = 2, *$*50,000–*$*99,999 = 3, and $100,000 and above = 4), race (white = 1), ethnicity (Hispanic = 1), and religion (Evangelical = 1). We also examine the influence of whether people get news from the social media (e.g., Facebook, Twitter), their concern about coronavirus (not at all concerned = 1, not very concerned = 2, somewhat concerned = 3, and very concerned = 4), direct experience with coronavirus (people or their family have been sick with coronavirus, no = 0, maybe = 0.5, yes = 1), and indirect experience with coronavirus (someone at work or others have been sick with coronavirus, no = 0, maybe = 0.5, yes = 1).

## Results and Discussion

**FIGURE 1:**
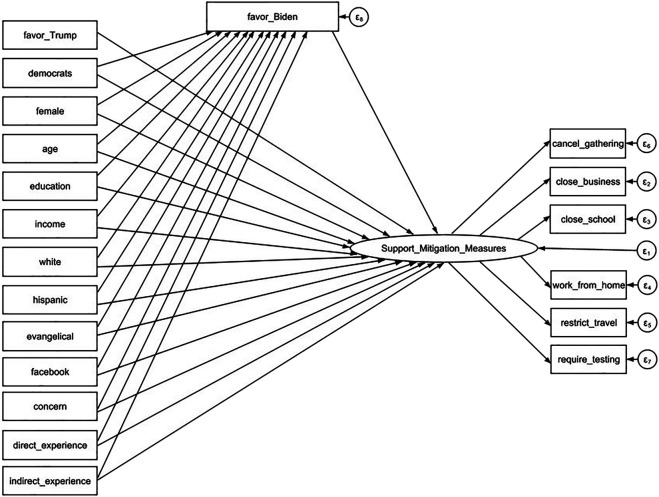
Structural Equation Modeling Diagram with Favorability of Biden as the Mediator. Understanding American Public Support for COVID-19 Risk Mitigation: The Role of Political Orientation, Socio-Demographic characteristics, Personal Concern, and Experience, the United States, 2020.

**FIGURE 2:**
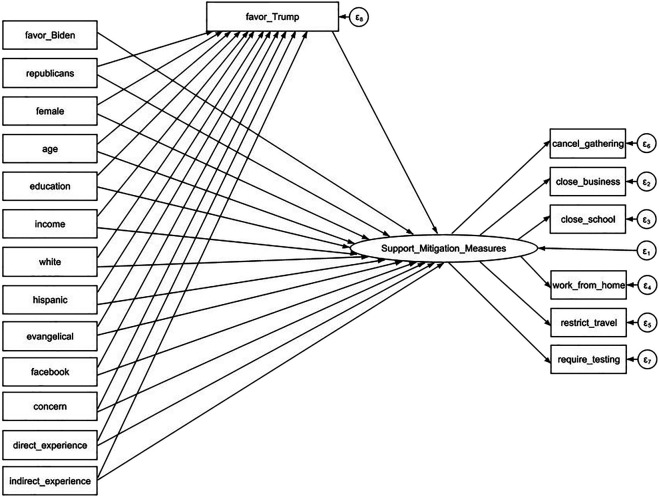
Structural Equation Modeling Diagram with Favorability of Trump as the Mediator. Understanding American Public Support for COVID-19 Risk Mitigation: The Role of Political Orientation, Socio-Demographic characteristics, Personal Concern, and Experience, the United States, 2020.

We estimate two SEM models with attitudes towards Biden and Trump as the mediator, respectively. The model using favorability of Biden as the mediator is presented in a diagram of Figure 1 and the model using favorability of Trump as the mediator is presented in a diagram of Figure 2. The exogenous variables including one’s political party affiliation, socio-demographic background have direct effects on their degree of support of mitigation measures. Meanwhile, these variables have indirect effects on the outcome variable channeled through the favorability variables. The results of standardized coefficients are reported in Table 3 and Table 4. We have weighted the data when performing SEM analysis by using the survey weight variable.

**TABLE 3:** Structural equation modeling results on public support for COVID-19 mitigation measures with favorability of Biden as the mediator. Understanding American Public Support for COVID-19 Risk Mitigation: The Role of Political Orientation, Socio-Demographic characteristics, Personal Concern, and Experience, the United States, 2020.

	Standardized coefficients	
	Direct effects	Indirect effects
Favorability of Biden	0.089***	—
Favorability of Trump	−0.124***	—
Democrats	0.041^†^	0.049***
Female	0.032^†^	−0.002
Age	0.020	0.003
Education	0.000	0.008**
Household Income	−0.010	−0.002
White	−0.019	−0.005*
Hispanic	0.024	0.002
Evangelical	0.030^†^	0.003^†^
Get news from the social media (e.g., Facebook, Twitter, etc.)	−0.026	0.002
Concern about coronavirus	0.535***	0.014***
Direct experience with the coronavirus	−0.003	0.003^†^
Indirect experience with the coronavirus	0.034*	−0.002

Note: †*p* < 0.1; *p < 0.05: **p < 0.01; *** < 0.001.

**TABLE 4:** Structural equation modeling results on public support for COVID-19 mitigation measures with favorability of Trump as the mediator. Understanding American Public Support for COVID-19 Risk Mitigation: The Role of Political Orientation, Socio-Demographic characteristics, Personal Concern, and Experience, the United States, 2020.

	Standardized coefficients	
	Direct effects	Indirect effects
Favorability of Trump	−0.124***	—
Favorability of Biden	0.089***	—
Republicans	−0.041^†^	−0.074***
Female	0.032^†^	0.006**
Age	0.020	−0.004^†^
Education	0.000	0.002
Household Income	−0.010	0.001
White	−0.019	−0.009**
Hispanic	0.024	−0.003
Evangelical	0.030^†^	−0.015***
Get news from social media (e.g., Facebook, Twitter, etc.)	−0.026	−0.003
Concern about coronavirus	0.535***	0.011***
Direct experience with the coronavirus	−0.003	−0.003
Indirect experience with the coronavirus	0.034*	0.003

†*p* < 0.1; *p < 0.05; **p < 0.01; ***p < 0.001.

The direct effects from either model suggest that attitudes towards these two political leaders have contrasting effects on public support for COVID-19 policies. Individuals who are in favor of Biden tend to express higher level of support for the mitigation policies, in comparison with those who are in favor of Trump. An early study finds that faith in Trump is a significant indicator of defying social distancing [9]. As Biden and Trump have consistently sent contrasting messages about COVID-19, their supporters have been influenced by their respective leaders’ views.

Because one’s favorability for political candidates reflects one’s underlying political ideology to a large extent, Republicans are found to be less likely to support COVID-19 mitigation measure than Democrats as Trump is the Republican leader and Biden is the Democratic leader. This finding is consistent with an earlier result showing that conservatives perceive lower level of risks of COVID-19 than liberals [2]. At an aggregate level, geographic areas with more Republicans are less likely to engage in social distancing measures compared to areas with more Democrats [3, 2]. This result confirms our first hypothesis.

The strong effect of elite cues further manifests itself in slanting public support of COVID-19 mitigation measures within the same party. As presented in Table 3, the coefficient for the indirect effect of the Democrats variable is positive and statistically significant (*β* = 0.049, *p* < 0.001). Thus, for Democrats, they are more likely to express support for COVID-19 measures if they are more in favor of Biden compared to their fellow Democrats who are less in favor of Biden. In contrast, as displayed in Table 4, the coefficient for the indirect effect of the Republicans variable is negative and statistically significant (*β* = −0.074, *p* < 0.001). For Republicans, they are less likely to show support for COVID-19 measures if they are more in favor of Trump, in comparison with their fellow Republicans who are less in favor of Trump. These two findings confirm our second hypothesis.

In addition to the results related to political factors, we find that gender and religious identity (Evangelical Christian) are significant factors determining public support for COVID-19 mitigation measures. Specifically, women are more likely than men to support these measures. This finding is different from earlier results as previous studies do not find significant differences existing between men and women in perceiving risks of COVID-19 [2] and in social distancing behavior [4] in their full models. It is however consistent with the finding of a recent study which shows that women are more likely than men to wear masks in 10 states in the United States [42]. A long line of research shows that women are more likely to express concerns for environmental and health hazards [43], possibly due to the biological differences between men and women (women are more vulnerable than men to environmental threats) [44] as well as different societal roles (women are more likely to be care providers) [45]. Because this direct effect of gender is significant at 0.1 level, we caution that more studies need to conducted to further investigate the role of gender in risk perceptions and risk mitigation behaviors associated with COVID-19. More interestingly, favorability towards Biden does not slant support for COVID-19 measures among women, whereas favorability towards Trump does. Women are less likely than men to show favor of Trump. Among women, those who are less in favor of Trump are more likely to support COVID-19 measures. Evangelical Christians are more likely than other Americans to support these measures. However, their favorability towards Trump can dampen their support. Evangelical Christians are one of the groups that have shown consistent support for Trump over the years despite the decrease of support in recent years [46]. Trump’s framing of various issues has undoubtedly exerted significant influence shaping Evangelicals’ views as the finding clearly suggests. The test of significance for the direct effect of evangelical is at 0.1 level. We thus caution that any over interpretation of this positive result may be subject to inaccuracy. Still, the effect of Evangelical Christian on support for these measures is intriguing, requiring rigorous theoretical examination and extensive empirical investigation.

Interestingly, using social media as news source does not have significant effects on support for COVID-19 mitigation measures. The non-significant effects of race and ethnicity are somewhat surprising, given earlier findings that white American adults perceive lower level of risks associated with COVID-19 than other racial groups and Hispanics perceive higher level of risks than other ethnic groups. It seems that varying levels of risk perceptions embodied by different racial/ethnical groups have not totally transferred to support for the risk reduction restrictive policies. We suspect it is partially due to the fact that we include concern for COVID-19 in the models. This variable is found to have the most significant effects. More details about the effects of this variable can be found in the following paragraph. Concern is a global construct of multidimensionality of risk perceptions. It is likely that all the racial/ethnical differences are manifested in the varying levels of concern which directly influence the support for these restrictive measures. Meanwhile, it is worth noting that risk perceptions can be conducive to risk reduction behaviors/behavioral intentions nevertheless cannot guarantee risk perception surely lead to risk reduction behaviors/behavioral intention. For instance, despite that black Americans are at higher level of risks associated with COVID-19, they are consistently less likely to say they would get a vaccine [47]. More research needs to be conducted to further investigate the racial/ethnic effects on support for COVID-19 risk reduction measures.

The most significant direct effects come from concern about COVID-19. Those who are more concerned about the pandemic are more likely to be in favor of Biden and express support for the mitigation measures. Further, among those who are concerned about COVID-19, their favorability of Biden can intensify their support. Symmetrically, people who are more concerned about the pandemic are less likely to be in favor of Trump. Moreover, the less favorable they feel toward Trump, the more likely they are to support these mitigation measures.

Direct experience with the coronavirus does not have any direct effects on support for mitigation measure. Interestingly, indirect experience with the coronavirus (co-workers and others in one’s social circle have been sick with this disease) has positive effects on support for mitigation. Our interpretation of the different impacts from direct and indirect experience is as follows. First, the number of people with indirect experience is almost as twice as that of those with direct experience. Impact of COVID-19 tends to reach people through others. One is much more likely to hear about the detrimental effects from those in one’s social circle. Second, given the relatively low mortality rate, those who have been sick with this disease are most likely to recover. The recovery provides one with not only physical immunity but also mental strength, to use Nietzsche’s famous quote “That which does not kill us makes us stronger.” Third, related to the second explanation, this finding may reflect the influence of self-interest in public health behaviors to some extent. If one has been sick with COVID-19 and developed immunity as a result, one would feel safe and less necessary to support these COVID-19 mitigation measures. If one’s experience is gained indirectly (e.g., hearing “horror stories” from others), one would be more concerned as uncertainty and suspense are still attached to COVID-19.

### Conclusion

A rapidly growing research has revealed a politically driven cleavage existing in American public response to COVID-19 in terms of risk perceptions and engaging in social-distancing behaviors. Drawing insights from the literature on framing theory, elite cues, construal level theory, and the Protective Action Decision Model, this study contributes to this research agenda by examining how political attitudes, socio-demographic characteristics, concern for COVID-19 and experience with the pandemic influence public support for a series of COVID-19 mitigation measures. All our four hypotheses are confirmed. First, we find evidence for strong effects of elite cues in influencing public support for relevant measures to reduce the risks posed by COVID-19. Specifically, Democrats are more likely than Republicans to show support for these measures. Further, Republicans who are more in favor of Trump are less likely to express support for COVID-19 mitigation measures, compared to their fellow Republicans who are less in favor of Trump. Symmetrically, Democrats who are more in favor of Biden are more likely to show support, in comparison with their fellow Democrats who are less in favor of Biden. The theoretical implication is that the effects of elite cues are further strengthened in a highly competitive framing environment. People form their opinions based on the combination of pre-existing ideology and new information [18]. Given the diversity of today’s information ecosystem, it is no longer plausible to shape public opinion *via* a uniform message. Since the early stage of this pandemic, President Trump and Democratic leaders have sent divergent messages regarding COVID-19 through their respective platforms and outlets. Their supporters and followers correspondingly form divergent perceptions and adopt different behavioral responses to this disease. Findings of this study once again highlight the enormous challenges of adopting nationally uniform measures to mitigate risks of COVID-19 in a deeply divided nation. Another highlight of this study is the finding on the different impacts of direct and indirect experience with the coronavirus. People with direct experience (either they or their family were sick with COVID-19) are not more likely than others to support the mitigation measures. Meanwhile, indirect experience (their co-workers or others in the social circle were sick with COVID-19) however motivates one to support mitigation measures. This finding poses an “experience paradox.” Personal experience in an indirect manner can shorten the psychological distance rendering the pandemic an imminent threat. Direct personal experience somehow leaves the impression that the pandemic can be overcome and no restrictions need to be imposed. It is also possible the direct experience can lead to the outcome of being immune therefore safe, rendering restrictions unnecessary. Given that the impact of personal experience on behavioral response is unsettled in the literature, we caution more follow-up studies need to be conducted to validate the finding of this study. Last but not least, concern for COVID-19 is found to be a strong factor on public support for mitigation measures, confirming the explanatory power of the global construct of multidimensionality of risk perceptions. Future studies should consider developing a list of items each of which represents various aspects of risk perceptions to further investigate the relationship between risk perceptions and risk mitigation behavioral response.
